# Discrete-Time Impedance Control for Dynamic Response Regulation of Parallel Soft Robots

**DOI:** 10.3390/biomimetics9060323

**Published:** 2024-05-28

**Authors:** Ameer Hamza Khan, Shuai Li

**Affiliations:** 1 Smart City Research Institute (SCRI), Hong Kong Polytechnic University, Kowloon, Hong Kong; ameer.h.khan@connect.polyu.hk; 2 Department of Land Surveying and Geo-Informatics (LSGI), Hong Kong Polytechnic University, Kowloon, Hong Kong; 3 Faculty of Information Technology and Electrical Engineering (ITEE), University of Oulu, 90570 Oulu, Finland

**Keywords:** soft robots, sliding mode, impedance control

## Abstract

Accurately controlling the dynamic response and suppression of undesirable dynamics such as overshoots and vibrations is a vital requirement for soft robots operating in industrial environments. Pneumatically actuated soft robots usually undergo large overshoots and significant vibrations when deactuated because of their highly flexible bodies. These large vibrations not only decrease the reliability and accuracy of the soft robot but also introduce undesirable characteristics in the system. For example, it increases the settling time and damages the body of the soft robot, compromising its structural integrity. The dynamic behavior of the soft robots on deactuation needs to be accurately controlled to increase their utility in real-world applications. The literature on pneumatic soft robots still does not sufficiently address the issue of suppressing undesirable vibrations. To address this issue, we propose the use of impedance control to regulate the dynamic response of pneumatic soft robots since the superiority of impedance control is already established for rigid robots. The soft robots are highly nonlinear systems; therefore, we formulated a nonlinear discrete sliding mode impedance controller to control the pneumatic soft robots. The formulation of the controller in discrete-time allows efficient implementation for a high-order system model without the need for state-observers. The simplification and efficiency of the proposed controller enable fast implementation of an embedded system. Unlike other works on pneumatic soft robots, the proposed controller does not require manual tuning of the controller parameters and automatically calculates the parameters based on the impedance value. To demonstrate the efficacy of the proposed controller, we used a 6-chambered parallel soft robot as an experimental platform. We presented the comparative results with an existing state-of-the-art controller in SMC control of pneumatic soft robots. The experiment results indicate that the proposed controller can effectively limit the amplitude of the undesirable vibrations.

## 1. Introduction

Soft robots, i.e., robots made from soft materials, have attained tremendous research attention in recent years [1,2,3,4,5]. They have shown great potential as a research platform as well as practical applications in industry [6,7] and healthcare [8,9,10,11]. Inspired by worms, snakes, and aquatic life [12,13], the main advantage offered by the soft robots are their flexible bodies, which impart them the ability to adapt to environmental variations automatically. They can easily navigate and interact [14,15] with previously unknown and irregular environments without any posing any danger to the interacting objects. Pneumatically driven soft robots, i.e., soft robots which use air pressure for actuation [16,17,18,19,20], have found most applications as compared to other soft robots because of their high flexibility, agility, and ease of fabrication [21]. This quality makes the pneumatic soft robots ideal for industrial applications [17,22,23] where safe handling of delicate objects is needed, as compared to rigid robots [24].

**Figure 1:**
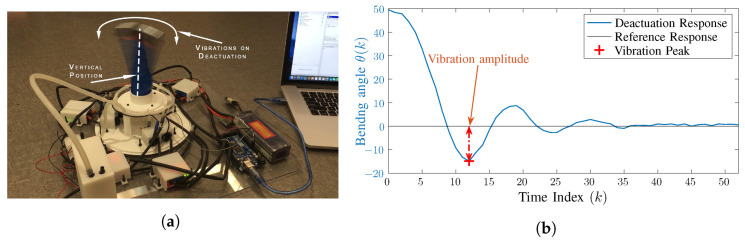
The vibration problem in soft robots. (**a**) shows the image of vibration amplitude, and (**b**) shows the response in the form of a graph. (Sampling Time: $T_{s}=0.02$).

However, despite their advantages, the flexible structure of soft robots also contributes to some of its inherent weaknesses. Foremost among those weaknesses is the lack of stiffness, which results in undesirable dynamic, i.e., vigorous vibrations and overshooting when actuated or deactuated rapidly as pointed by [25,26]. These undesirable dynamics increases the overall settling time of the pneumatic soft robots, which not only decrease their accuracy and productivity but also reduce their useful lifespan by creating structural defects in their body. Figure 1 illustrates the problem of excessive overshooting and vibrations in soft robots. In the figure, the soft robot undergoes a vibration amplitude of about 50 degrees and a settling time of about 0.8 s. These dynamics can create the following issues in industrial applications

- Large settling time reduces productivity.
- Large amplitude oscillations reduce accuracy and can potentially cause damage to a delicate product.
- Oscillations accelerate the process of wear and tear in soft robots, thus increasing maintenance costs.

These issues are inhibiting the large-scale adaption of soft robots in industrial applications. Most of the industrial applications require the soft robot to operate at an increased speed with a reasonable degree of accuracy and robustness [27]. In order to increase the utility of the soft robots in industrial applications, the issues posed by vibrations, overshooting, and large settling time need to be addressed.

Several approaches have been proposed in soft robotic literature to control the motion of soft robots. Finite Element Method (FEM) [28] is a popular technique for the kinematic control of soft robots. However, such methods require high computation cost and cannot be realistically used for controlling the dynamic response in real-time. The use of a model-free controller such as PI [29] has also been investigated for the control of pneumatic soft robots. However, in the presence of model nonlinearities, the tuning of PI parameters becomes a time-consuming and labor-intensive task. Passive approaches have been proposed in the literature to regulate the dynamic behavior of the pneumatic soft robots by using the natural damping properties of the materials [30]. For example, Ni et al. [25] propose attaching an external damper to the soft robot, while Li et al. [26] propose particle damping approach by attaching an additional grain-filled chamber to the soft robot. These external components help in regulating the dynamic behavior by dissipating excessive kinetic energy. Although these methods effectively suppress the oscillations, but at the cost of attaching the additional mechanical component. This mechanical overhead makes the system heavy, bulky, and less portable and increases the overall fabrication cost of soft robots. Luo et al. [31] make further progress in this direction by proposing a Sliding-Mode Controller (SMC) to regulate the dynamic response; however, their control law is essentially a PD controller with a deadband and does not account for external disturbances. Similar to the PI controller proposed by [29], their proposed SMC controller relies heavily on manual parameter tuning. Additionally, once tuned, the controller parameters remain constant and do not adapt to variation in the soft robot. Soft robots undergo inevitable wear and tear [32] after several usage cycles, which primarily impact the model of soft robots. The controllers mentioned above are non-adaptive to the model variations, which results in gradual degrading of dynamic response.

In this paper, we propose a novel algorithm to regulate the dynamic characteristics of the soft robot by using impedance control [33,34,35,36,37] approach. The impedance control is used to simultaneously regulate both the position and force in a mechanical system. By controlling the impedance of a mechanical system, the required dynamic response can be achieved. In order to enforce the required impedance on the pneumatic soft robot, a nonlinear sliding mode controller (SMC) [1,31,36] is proposed in this paper. The sliding mode surface for the controller is defined according to the desired dynamic response, i.e., on the sliding surface system, the impedance of the system closely matches the desired impedance. Due to model irregularities and nonlinearities, the system can quickly diverge from the sliding surface. Therefore, a nonlinear switching control action term [38,39,40] is added to the controller, which always drives the system toward the sliding surface and guarantees its convergence. Additionally, the proposed SMC is discretized to avoid state observers [41], which significantly reduces the computational load. The discrete controller can easily be extended to higher-order systems and efficiently implemented on embedded processors.

The novelty of our proposed controller is the ability to achieve the desired dynamic response by directly specifying the impedance parameters instead of relying on manual parameter tuning. Our proposed controller uses the identified model of the robot to calculate the controller parameter automatically. To the best of our knowledge, no literature work related to soft pneumatic robot focuses on the impedance control to regulate their dynamic response. The main contributions of this paper can be summarized as follows

- Formulation of a discrete sliding mode controller based on impedance control for regulating the dynamic response of soft robots, suppressing undesirable dynamics and reducing settling time.
- Automatic calculation of the controller parameters based on the required impedance of the soft robot. It removes the reliance on a human operator to manually tune the controller parameters as required by the traditional controller.
- Formulation of the proposed sliding mode controller in discrete time to avoid the state observers. It reduces the computational complexity and makes the controller easily extendable to higher order systems models.

The remainder of this paper is distributed as follow. The impedance control problem is formally stated in Section 2, and then we propose the formulation of the SMC controller in Section 3. Later in Section 4, we present the design of our experimental platform used to test the proposed SMC controller on the soft parallel robot. Section 5 presents the experimental results and Section 6 concludes the paper.

## 2. Problem Formulation

In this section, we will present the formal mathematical definition of the impedance control problem.

### 2.1. Dynamic Model

The Sliding Mode Controller (SMC) is based on the dynamic model of the system. Consequently, it requires the construction of state observers for the estimation of higher-order terms in the system model. The computational burden and complexity of state observers can be avoided by formulating the model and controller in discrete-time. In this paper, we will formulate a generalized discrete SMC, which can easily be extended to high order systems models. The generalized discrete-time transfer function of a practical system, whose next state depends only on the present and past values of state and input can be written as follows,

(1) $$H\left(z\right)=\frac{\theta (z)}{U(z)}=\frac{b_{1}z^{-1}+b_{2}z^{-2}+...+b_{m}z^{-m}}{a_{0}+a_{1}z^{-1}+...+a_{n}z^{-n}},$$

where θ is the bending angle of our soft robot, and *u* is the input air pressure. The above discrete transfer function is equivalent to the following state-space model with added component d(k) for modeling errors and disturbances,

(2) $$\theta \left(k+1\right)=\sum_{i=0}^{n-1}A_{i}\theta \left(k-i\right)+\sum_{j=0}^{m-1}B_{j}u\left(k-j\right)+d\left(k\right)$$

where $A_{i}=-a_{i+1}/a_{o}$ and $B_{j}=b_{j+1}\cdot a_{i}$ and $b_{i}$ are the model parameters. The above equation represents an n-th order system model. Based on the perturbation method, we can generate an approximation of d(k) during runtime, following the approach of [34,41,42,43,44], using one-step delayed estimation as follow

(3) $$\begin{array}{cc} \hat{d}\left(k\right) & =d(k-1)\\ & =\theta \left(k\right)-\sum_{i=0}^{n-1}A_{i}\theta \left(k-i-1\right)-\sum_{j=0}^{m-1}B_{j}u\left(k-j-1\right). \end{array}$$

where $\hat{d}\left(k\right)$ denotes an estimation of the real disturbance value d(k). Using the above estimation, the dynamic model in (2) can be written as,

(4) $$\begin{array}{c} \theta \left(k+1\right)=\sum_{i=0}^{n-1}A_{i}\theta \left(k-i\right)+\sum_{j=0}^{m-1}B_{j}u\left(k-j\right)+\hat{d}\left(k\right)+\tilde{d}\left(k\right), \end{array}$$

where $\tilde{d}\left(k\right)=d\left(k\right)-\hat{d}\left(k\right)$ is the disturbance estimation error.

**Assumption** **A1.** *The rate of change in disturbance d(k) is bounded i.e., $|\dot{d}\left(t\right)|<\rho$ for some constant value of ρ. This can be discretized using Euler backward approximation method as follow,*

$$|\dot{d}\left(t\right)|\approx \left|\frac{d(k+1)-d(k)}{T_{s}}\right|\leq \rho ,$$

*where $T_{s}$ is the sampling time. The above equation can be further written as,*

(5) $$\begin{array}{c} \left|d\left(k+1\right)-d\left(k\right)\right|\leq \rho T_{s}. \end{array}$$

This relation mathematically models the assumption that the maximum difference between current and the future value of real disturbance d(k) depends on sampling time $T_{s}$ and bounded by a maximum value of $\rho T_{s}$. This assumption will be used later in the stability analysis of the controller.

### 2.2. Impedance Control

The relation between system motion and the applied force is defined as the impedance of the system. Impedance control deals with the simultaneous control of motion as well as the force to regulate the dynamic impedance of the system [35]. By appropriately regulating the force-motion relations of a system, the dynamic response of the system, i.e., overshoot, settling time, and vibrations, can be accurately controlled. Now we will formally define the impedance control problem.

Let *f* be the force being applied to the soft robot. The force will generate some bending motion θ in the soft robot. By following the strategy of [34,45,46] the generalized impedance model for the trajectory tracking of the soft robot can be derived as [33,35,47,48,49]

(6) $$m{\ddot{e}}_{\theta }\left(t\right)+b{\dot{e}}_{\theta }\left(t\right)+ke_{\theta }\left(t\right)=k_{f}f\left(t\right),$$

where $e_{\theta }\left(t\right)=\theta \left(t\right)-\theta_{r}\left(t\right)$ is the position error. $\theta_{r}\left(t\right)$ denotes the desired bending angle, also called reference signal. The *m*, *b*, *k* and $k_{f}$ are the design parameters and chosen according to the required impedance. These parameters control the final dynamic response of the controlled system. The above equation can be discretized by using Euler backward difference as [34]

$$\begin{array}{cc} e_{\theta }\left(t\right) & =e_{\theta }\left(k+1\right),\;{\dot{e}}_{\theta }\left(t\right)=\frac{e_{\theta }\left(k+1\right)-e_{\theta }\left(k\right)}{T},\\ {\ddot{e}}_{\theta }\left(t\right) & =\frac{e_{\theta }\left(k+1\right)-2e_{\theta }\left(k\right)+e_{\theta }\left(k-1\right)}{T^{2}}. \end{array}$$

Using the above approximations with (6), the continuous impedance model can be discretized as follow,

(7) $$\begin{array}{c} Me_{\theta }\left(k-1\right)+Be_{\theta }\left(k\right)+Ke_{\theta }\left(k+1\right)=K_{f}f\left(k+1\right), \end{array}$$

where the parameters *M*, *B*, *K* and $K_{f}$ are defined as,

$$\begin{array}{c} M=\frac{m}{T^{2}},\;B=-\frac{2m}{T^{2}}-\frac{b}{T},\;K=\frac{m}{T^{2}}+\frac{b}{T}+k,\;K_{f}=k_{f}. \end{array}$$

If the system (2) is forced to follow the dynamics of the impedance model of (7), then the desired transient response will be achieved. However, in practical scenarios, because of the significant disturbance d(k), it is quite challenging to make the system follow the desired impedance response using a linear controller. The nonlinear discrete SMC solves this issue by using a nonlinear term in control law u(k), which guarantees that system closely follow the required dynamic response of (7). The convergence of system response to desired dynamic behavior will be proven in the next section.

## 3. Discrete SMC Formulation

In this section, we will first present the formulation of discrete SMC controller and then prove its stability under the influence of disturbances d(k).

### 3.1. Controller Design

The SMC is formulated by considering that the control objective is to enforce the dynamics of (7) on system (2). Therefore the sliding function [44,50,51] is defined as follows

(8) $$s\left(k\right)=c_{1}e_{\theta }\left(k-1\right)+c_{2}e_{\theta }\left(k\right)+c_{3}e_{\theta }\left(k+1\right)+c_{4}f\left(k+1\right),$$

where we take $c_{1}=M$, $c_{2}=B$, $c_{3}=K$ and $c_{4}=-K_{f}$.

It can be seen that if the sliding function s(k) converges to 0, then the system will produce the desired impedance behavior of (7). The SMC can help in achieving this objective by maintaining the system on the sliding surface s(k)=0, thus giving the desired impedance behavior. In the following, we will drive the SMC to reach the sliding surface in finite time and then prove its stability.

According to (8), the sliding surface s(k)=0 can be defined as,

(9) $$c_{1}e_{\theta }\left(k-1\right)+c_{2}e_{\theta }\left(k\right)+c_{3}e_{\theta }\left(k+1\right)+c_{4}f\left(k+1\right)=0.$$

Different definitions of discrete sliding mode are used in literature [52]. According to our definition of the sliding surface s(k), we will use s(k+1)=s(k)=0 in this paper. The resultant controller will try to direct the system toward the sliding surface in a single iteration. It results in faster convergence and robust performance.

Equation (9) can be rewritten as follows

(10) $$c_{1}e_{\theta }\left(k-1\right)+c_{2}e_{\theta }\left(k\right)+c_{3}\left\{\theta \left(k+1\right)-\theta_{r}\left(k+1\right)\right\}+c_{4}f\left(k+1\right)=0.$$

Since the control objective is to enforce the dynamics of above equation to the soft robot, we will replace the value of θ(k+1) from robot model (4) to the above equation. After algebraic manipulation and ignoring the unknown disturbance estimation error $\tilde{d}\left(k\right)$, the final expression for equivalent control action $u_{eq}\left(k\right)$ can be obtained as follow [34,42,48]

(11) $$\begin{array}{cc} u_{eq}\left(k\right)= & -B_{0}^{-1}\{\sum_{i=0}^{n-1}A_{i}\theta \left(k-i\right)+\sum_{j=1}^{m-1}B_{j}u\left(k-j\right)\\ & +\hat{d}\left(k\right)-\theta_{r}\left(k+1\right)\}\\ & -{\left(c_{3}B_{0}\right)}^{-1}\left\{c_{1}e_{\theta }\left(k-1\right)+c_{2}e_{\theta }\left(k\right)+c_{4}f\left(k+1\right)\right\}. \end{array}$$

The above control action will give the required dynamic performance if the disturbance estimation $\hat{d}\left(k\right)$ is ideal i.e., $\hat{d}\left(k\right)=d\left(k\right)$. This control action will keep the system on the sliding surface if it is initialized on the sliding surface, and there are no unknown external disturbances. If the initial starting point starts away from the sliding surface or if there a disturbance estimation error, i.e., $\hat{d}\left(k\right)\neq d\left(k\right)$, the equivalent control action cannot drive the system back to the sliding surface. A nonlinear switching control action is defined, to overcome this issue, as follows

(12) $$u_{sw}\left(k\right)=-G{\left(c_{3}B_{0}\right)}^{-1}\mathrm{sign}\left(s\left(k-1\right)\right),$$

where sign(.) is the signum function and *G* is a positive gain. Factor of ${\left(c_{3}B_{0}\right)}^{-1}$ is added to make notation simpler in the stability analysis, later in this section. The actual control action u(k) is defined as the combination of the equivalent control action $u_{eq}\left(k\right)$ and the nonlinear switching control $u_{sw}\left(k\right)$,

$$u\left(k\right)=u_{eq}\left(k\right)+u_{sw}\left(k\right).$$

By putting the values from (11) and (12), the final form of the control law becomes,

(13) $$\begin{array}{cc} u(k)= & -B_{0}^{-1}\{\sum_{i=0}^{n-1}A_{i}\theta \left(k-i\right)+\sum_{j=1}^{m-1}B_{j}u\left(k-j\right)\\ & +\hat{d}\left(k\right)-\theta_{r}\left(k+1\right)\}\\ & -{\left(c_{3}B_{0}\right)}^{-1}\left\{c_{1}e_{\theta }\left(k-1\right)+c_{2}e_{\theta }\left(k\right)+c_{4}f\left(k+1\right)\right\}\\ & -G{\left(c_{3}B_{0}\right)}^{-1}\mathrm{sign}\left(s\left(k-1\right)\right). \end{array}$$

### 3.2. Stability Analysis

Now we will show that the the controller (13) will drive the system (4) toward the sliding surface (9) in finite number of steps.

Replacing the value of u(k) from (13) into robot model (4) and after simplification, we get,

$$\begin{array}{cc} \theta (k+1)=\; & \theta_{r}\left(k+1\right)+\tilde{d}\left(k\right)-Gc_{3}^{-1}\mathrm{sign}\left(s\left(k-1\right)\right)\\ & -c_{3}^{-1}\left\{c_{1}e_{\theta }\left(k-1\right)+c_{2}e_{\theta }\left(k\right)+c_{4}e_{\theta }\left(k-1\right)\right\}. \end{array}$$

If we replace this value of θ(k+1) in (8) by using the $\theta \left(k+1\right)-\theta_{r}\left(k+1\right)=e_{\theta }\left(k+1\right)$, we get the following expression for the sliding function s(k) after simplification,

(14) $$\begin{array}{c} s\left(k\right)=-G\mathrm{sign}\left(s\left(k-1\right)\right)+c_{3}\tilde{d}\left(k\right). \end{array}$$

We now need to show that s(k) converges to the sliding surface i.e., s(k)=0 in finite time-steps. This can be shown by following the scheme of [34]. Lets assume that $G=K_{3}\left|\tilde{d}\left(k\right)\right|+\sigma$, where σ is an arbitrary positive constant. Based on such value of *G* and (14), the following conclusion can be drawn,

$$\sigma <|s\left(k\right)|<2c_{3}\left|\tilde{d}\left(k\right)\right|+\sigma .$$

According to (5) as stated in assumption 1, the disturbance is bounded

$$|\tilde{d}\left(k\right)|\leq \rho T,$$

therefore, we have the following bound on the value of s(k),

$$\sigma <|s\left(k\right)|<2c_{3}\rho T+\sigma .$$

This shows that the value of s(k) will reach in $2c_{3}\rho T+\sigma$ neighborhood of sliding surface s(k)=0 within finite number of steps.

**Remark** **1.** *The sign(s) function is a discontinuous function at s=0. The discontinuity introduces a chattering in the control input u(k). The chattering can be avoided by gradually changing the value of the switching function. There are several ways to smooth the function as shown by [38,39,40,53]. For our controller, we used the saturation function [34] for its simplicity and computational efficiency*

(15) $$\begin{array}{c} \mathrm{sat}\left(s\right)=\left\{\begin{array}{cc} +1, & \mathrm{if}\;s>+\delta \\ s/\delta , & \mathrm{if}\;-\delta <s<+\delta \\ -1, & \mathrm{if}\;s<-\delta \end{array}\right. \end{array}$$

*where δ corresponds to the width of the ramp in saturation function.*

## 4. Experimental Platform

In this section, we will first describe the fabrication, actuation, sensing, and control mechanism for our soft robot and then present the system identification method for estimating the parameters $A_{i}$’s and $B_{i}$’s in (4) for our soft robot.

### 4.1. Soft Parallel Robot

#### 4.1.1. Design and Fabrication

**Figure 2:**
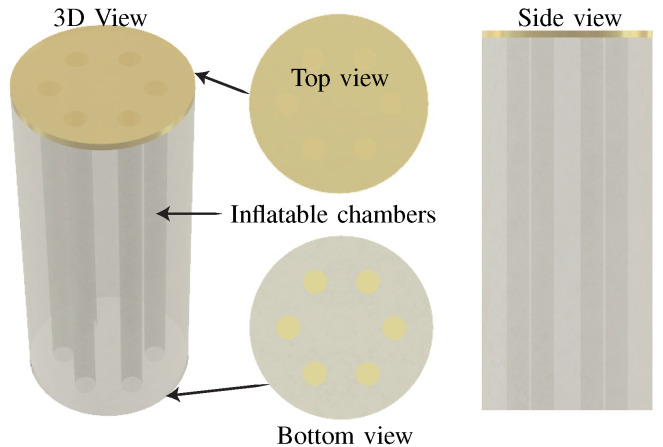
3D model of the soft robot. Left: 3d view, middle-up: top view, middle-down: bottom view, and right: side view of the soft robot.

For experiments, we used a pneumatically actuated soft parallel robot, as shown in Figure 2. The soft robot consists of a cylindrical body, and 6-parallel inflatable chambers radially distributed inside its body. The soft robot is capable of bending motion when actuated using pressurized air. Since the soft robot consists of 6 inflatable chambers, it is capable of performing a motion in 3D-space.

We designed the molds for the fabrication of our soft parallel robot. The molds were prepared using a 3D printer. We chose Ecoflex-30 (Smooth-On Inc., Macungie, PA, USA) silicone material as the fabrication material of our soft robot because of its high flexibility and excellent strength. Ecoflex-30 consists of two separate liquid components. The liquid silicone mixtures were prepared by mixing both components in equal proportion. The mixture was then poured in the 3d-printed molds and left for curing at atmospheric temperature and pressure for about 8 h. After the curing of silicone is complete, the soft robot was removed from the molds.

#### 4.1.2. Actuation and Sensing

**Figure 3:**
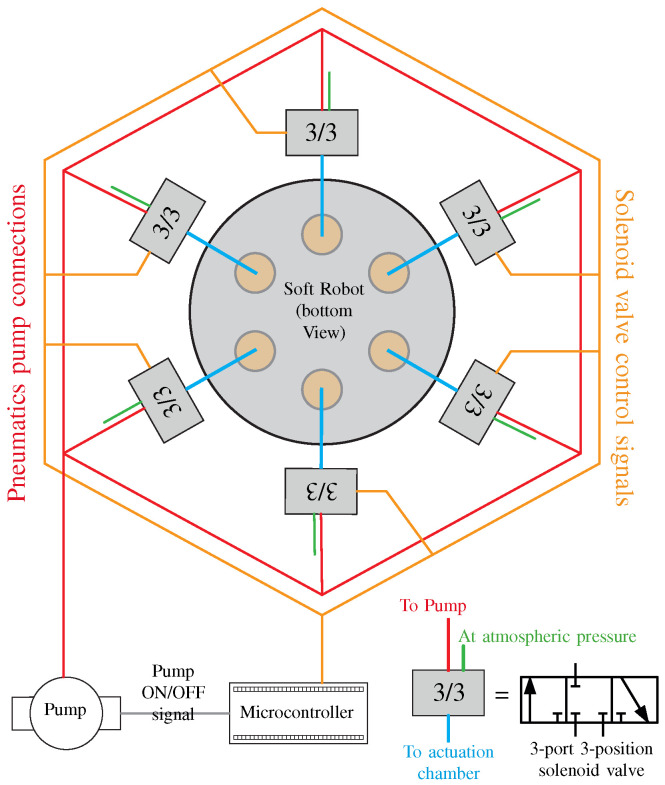
Schematic diagram of the actuation mechanism and control components used in the experimental platform for our soft parallel robot.

Our soft parallel robot is based on pneumatics and uses air pressure for the actuation of inflatable chambers. To provide the necessary air pressure, we used a 12V-DC vacuum-pump. Since the input u(k) to the soft robot is the air pressure, therefore we also used pneumatic- valves to regulate the air pressure from the pump to the inflatable chambers of the soft robot. The schematic diagram of the actuation mechanism is shown in Figure 3. A 3-port 3-position valves connected between each chamber and the vacuum pump. A 3-port 3-state valve can exist in 3 states: state one corresponds to airflow from pump to chambers, state 2 corresponds to holding the air inside the chamber, and position three is used to direct airflow out from the chambers to the atmosphere. By controlling the valve states, the air pressure inside the chambers can be controlled as required by the control law u(k).

The valves require high electric power for switching between state, which cannot be directly provided by the embedded microcontrollers. To overcome this issue, we used MOSFET switches between the valves and the microcontrollers. The MOSFET switches have a high power rating and fast switching speed, which makes them ideal for controlling the valve states.

**Figure 4:**
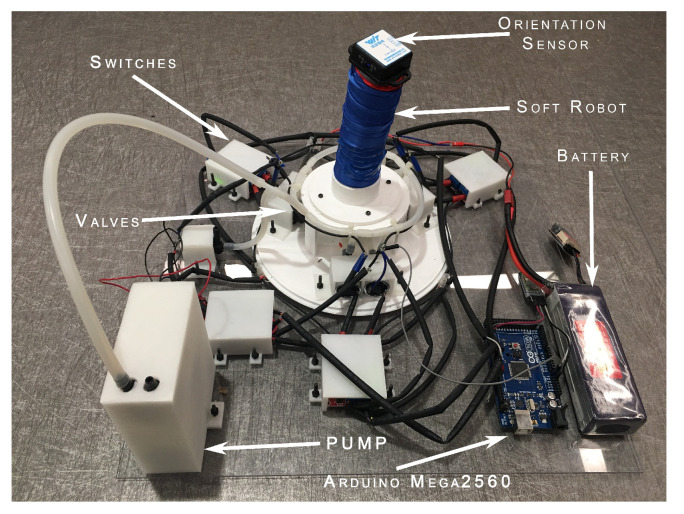
Experimental platform to test the effectiveness of the proposed controller for our soft parallel robot. All the major components are labeled in the image.

Our soft robot is capable of performing bending motion in the 3D-space. Therefore, we used an orientation sensor to measure its bending motion. The orientation sensor is mounted on the top surface of the soft robot, as shown in Figure 4. The orientation sensor gives the magnitude of the bending angle relative to the horizontal top surface of the soft robot. In the normal state, when the soft robot is the vertical position, the orientation gives zero angles of bending. When a bending motion is created in soft robot and the top surface orient at an angle relative to the horizontal plane. The mounted sensor provides this magnitude of this orientation angle. The sensor is connected to the microcontroller wirelessly using a Bluetooth module. The wireless sensing can even allow a separate server for data acquisition and processing.

#### 4.1.3. Controller Implementation

For the digital implementation of the discrete SMC controller, we used an Arduino Mega2560 microcontroller (RS Components, Hong Kong). We used an additional HC-05 Bluetooth module for wireless connection with the orientation sensor using the microcontroller’s serial port. The digital pins of the microcontroller are connected with the MOSFET switches to drive the pneumatic valves. The high clock speed provided by the Arduino mega2560 microcontroller provides a reliable solution for real-time data acquisition and processing for the SMC control algorithm.

**Figure 5:**
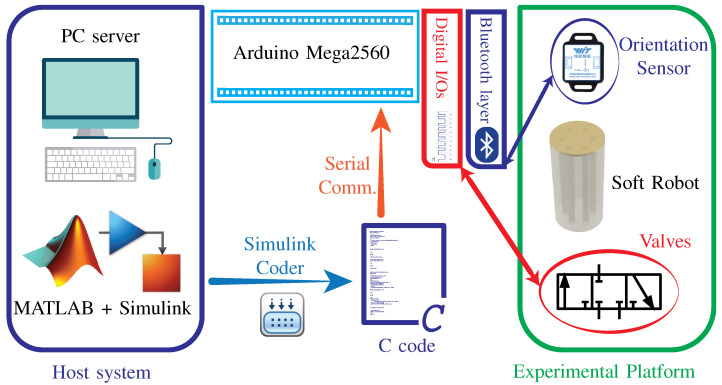
Schematic diagram of the control architecture used in experiments. It shows the interaction of software and hardware components of the platform.

The commonly used approaches for implementing a controller on an embedded processor requires writing the C code. This approach is prone to bugs and increases the implementation time. To increase the implementation speed and improve the reliability of the implemented control algorithm, we used Simulink to design and test our control algorithm. Once the controller design is tested and finalized, we used Simulink Coder to generate C code for the Arduino mega2560 target automatically. This approach offers several advantages over manually writing the C code. Simulink is widely accepted as an excellent platform for control design, simulation, and testing [54]. Combining it with the Simulink coder allows for rapid prototyping and testing of the control algorithm while avoiding the complexity and bugs of the underlying C code. This increases the speed and reliability of the controller implementation stage. The sampling time ($T_{s}$) of the control loop was set at 0.02 s. The schematic diagram of the controller implementation is shown in Figure 5.

### 4.2. Model Identification

**Figure 6:**
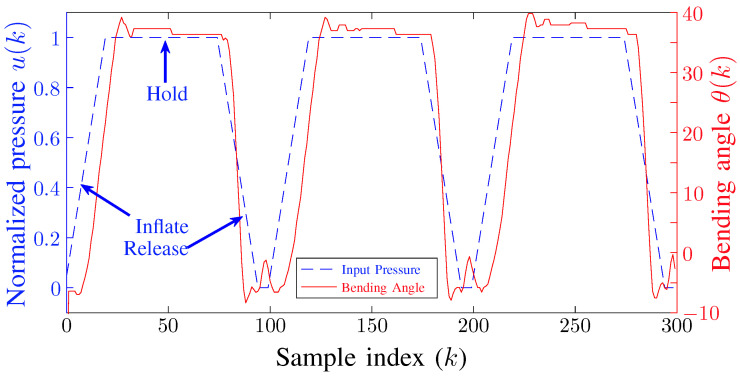
System response for inflate-hold-release experiment used to estimate the robot’s model parameters $A_{i}$’s and $B_{i}$’s according to (4). (Sampling Time: $T_{s}=0.02$).

After the construction of the experimental platform for our soft parallel robot, our next task was to estimate the model of the soft robot according to system model (4). The objective of this task was to estimate the model parameters $A_{i}$’s and $B_{i}$’s. Since all the chambers are identical, therefore we only need to run the estimation experiment for a single chamber. The disturbance estimation $\hat{d}\left(k\right)$ will account for minor differences between chambers. To estimate the parameters, we repeated the inflate-hold-deflate experiment with a time-period of 2 s and recorded the bending angles of the soft robot. During the experiment, a valve was switched to state 1 to apply high-pressure air to the corresponding chamber of the soft robot, then the valve was switched to state 2 to maintain the air pressure, and at the end, the valve was switched to state 3 to release the air pressure. The measured system response is shown in Figure 6. Once the input-output data was collected, we used MATLAB (R2022a) system identification toolbox (R2022a) [55] to estimate the model parameters. We estimated a 3rd order system model because its calculated response closely matched the measured response.

## 5. Simulations and Experiments

In this section, we will present the experimental methodology and the obtained results. The experimental results show the efficacy of the presented SMC control algorithm in regulating the dynamic response and suppress the vibration amplitude. We will first present the simulation results and then present the results of experiments on our experimental platform.

### 5.1. Simulations

We first conducted a simulation-based evaluation of the discrete SMC to verify the effectiveness of the proposed algorithms. The simulation results help in assessing the stability and safety of the controller before trying it on the experimental platform. We used Simulink [54] as the simulation environment since later, we can directly use the simulation files for hardware implementation using the Simulink Coder (R2022a). We conducted a series of simulations using different system models, impedance parameters, and initial conditions.

**Figure 7:**
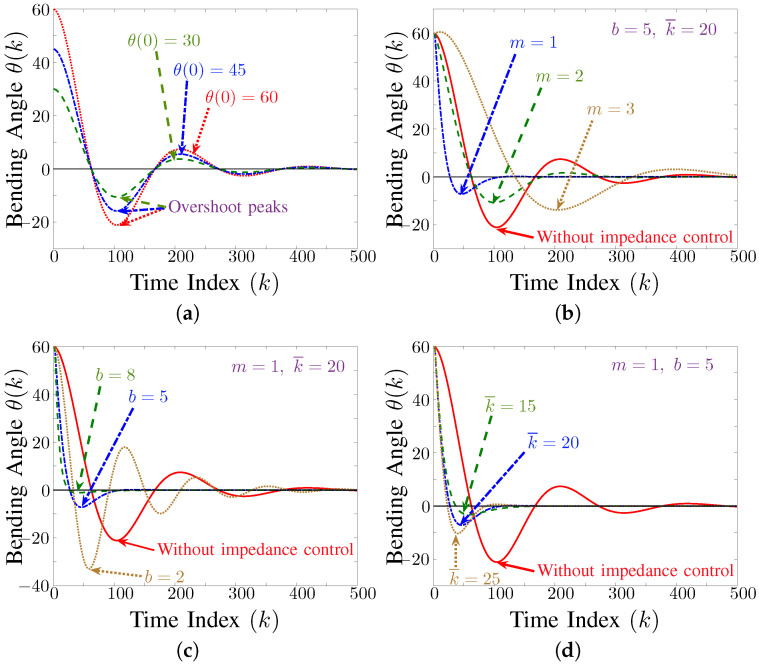
Simulation results showing impact of parameter variation on the settling time and vibration amplitude. (**a**) shows the deactuation response of the system for different initial conditions without any impedance control. (**b**–**d**) shows the impact of variation in parameter *m*, *b*, and $\bar{k}$ on system response. (Sampling Time: $T_{s}=0.02$).

One of the important choices for the impedance control was the selection of vibration damping force f(t) profile along the robot trajectory, according to (6). For the purpose of simulation and experimentation, we chose the damping force to be proportional to the position error i.e., $f\left(t\right)=ce_{\theta }\left(t\right)$. It allows a large vibration-damping force when the soft robot is far from the vertical position, which gradually decreases as the robot return to the vertical position. The equivalent impedance equation can be written as,

(16) $$m{\ddot{e}}_{\theta }\left(t\right)+b{\dot{e}}_{\theta }\left(t\right)+\bar{k}e_{\theta }\left(t\right)=0,$$

where $\bar{k}=k-ck_{f}$. The results of varying the parameters *m*, *b*, and $\bar{k}$ are shown in Figure 7. The impact of different parameter values on the settling time and the vibration amplitude can be seen. Figure 7a shows that increasing the value of parameter *m* increases both the settling time and the vibration amplitude; thus, it is desirable to decrease the value of *m*. However, its lower value is bounded by the actuator’s capacity, i.e., pump and valves speed, and cannot be made infinitesimally small. Similarly, it can be observed in Figure 7c that increasing *b* decreases the vibration amplitude. Figure 7d shows that increasing $\bar{k}$ will correspondingly increases the vibration amplitude. These correlations between values of *m*, *b*, and $\bar{k}$ also matches with the theoretical analysis of the second-order impedance Equation (7).

### 5.2. Experimental Results

**Figure 8:**
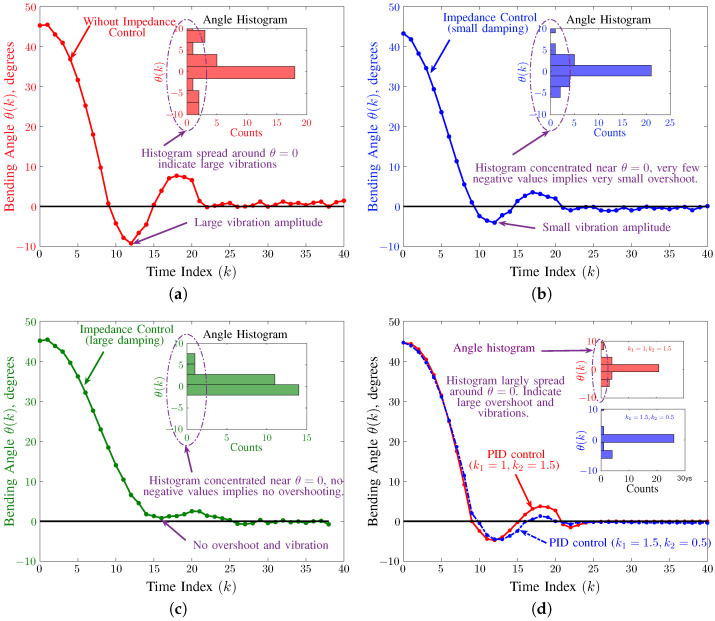
The experimental results, showing the performance of the proposed impedance control scheme. (**a**) the performance without the impedance control, shows large overshoot and vibrations. (**b**) the performance with low impedance ($m=1,\;b=20,\;\bar{k}=500$), shows small overshoot. (**c**) the performance for high impedance ($m=1,\;b=25,\;\bar{k}=500$) with almost no overshoot and minimal vibration. (**d**) The performance of PID controller in (17) for two different values of parameters $k_{1}$ and $k_{2}$, overshooting similar to (**b**). (Sampling Time: $T_{s}=0.02$).

We conducted experiments on our soft robot to demonstrate the effectiveness and performance of the proposed discrete SMC. These experiments check the performance of the controller on the deactuation of the soft robots since most vibrations are created on deactuation. The first set of experiments consists of a single axis motion of the soft robot in which one of the chambers is initially actuated with air and then deactuated using impedance control. The comparison for three experimental trials is shown in Figure 8. Figure 8a shows the performance of the soft robot without using impedance control. It can be seen that there is a large overshoot and vibrations as the soft robot approaches reference angle θ=0. We then implemented the impedance control with values of impedance parameters be m=1, b=20 and $\bar{k}=500$. We chose these values based on the experimental trials and the estimated model of the soft robot. Figure 8b shows the results of using the impedance control on our soft robot. It can be seen that the vibration amplitude is smaller as compared to Figure 8a, which illustrates the effectiveness of the proposed SMC scheme in suppressing the vibration amplitude. In the next experiment, we modified the impedance parameters of the controller by changing b=25. As already shown in the simulation results, increasing the value of *b* increases the overall damping force in the system. The experimental result in Figure 8c also shows agreement with the simulation results. It can be seen that there is no overshooting in this case and minimal vibrations.

To further illustrate the difference between the three experimental cases, a histogram of angle values around θ=0 ([−10, 10]) is shown inside each plot. In the case of no impedance control, as shown in Figure 8a, the histogram is spread in the entire range from −10 to 10. The distribution of angle values indicates a large deviation from θ=0. In the second case, when the damping force is small, as shown in Figure 8b, the angle values are concentrated around θ=0. Whereas in the third case, when damping force is large, as shown in Figure 8c, the angle values are again more concentrated around θ=0 with no data points below θ=0.

**Figure 9:**
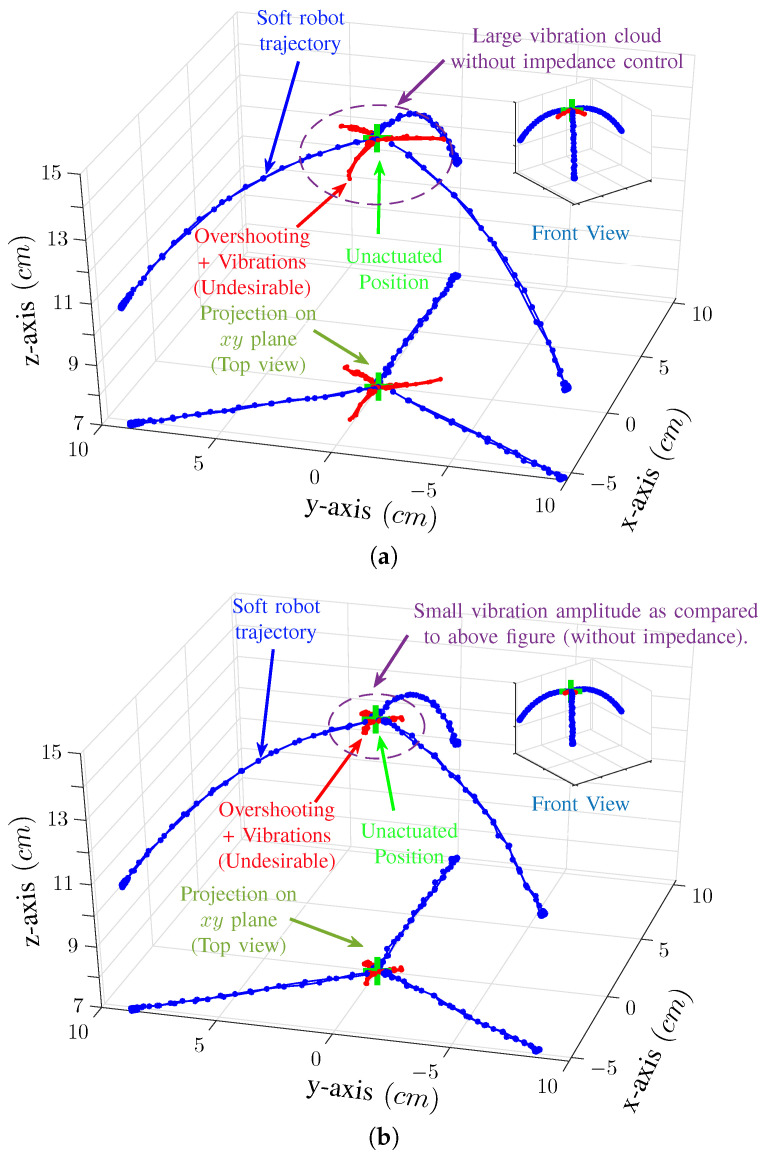
Illustration of 3D motion of the soft robot. Three chambers of the soft robot were actuated and then deactuated in consecutive turns. This figure shows a comparison between natural deactuation and impedance control. (**a**) shows result without the impedance control and (**b**) show response with impedance control. (Sampling Time: $T_{s}=0.02$).

The above-presented simulation and experimental results sufficiently demonstrate the efficacy of the proposed discrete SMC based impedance control scheme. We then ran a trajectory tracking experiment for our soft robot. In this experiment, three of the chambers of the soft robots were actuated and then deactuated sequentially using impedance control. Figure 9 shows the result of these experimental trails in the form of a 3D graph. Figure 9a shows the result without impedance control; it can be seen that the vibration (shown as red) have a large magnitude. In comparison, Figure 9b shows the results with the impedance control. It can be seen that the vibration cloud, in this case, is tiny as compared to Figure 9a.

### 5.3. Comparative Results

We conducted experiments to determine the comparative performance of the proposed controller with the state-of-art SMC dynamic response controller [31]. As mentioned in Section 1, their proposed continuous-time controller is essentially a PD controller with a deadband, which can be formulated as follow,

$$\begin{array}{c} \sigma \left(t\right)={\dot{e}}_{\theta }\left(t\right)+De_{\theta }\left(t\right) \end{array}$$

where *D* is the controller parameter need to be manually tuned. The input u(t)=σ(t) is only applied if |σ(t)|>ϵ, where ϵ corresponds to width of deadband. If σ(t)<0 then u(t)=0. Based on their strategy, we used the following general structure of PID controller in our experiments,

$$\begin{array}{c} \sigma \left(t\right)={\dot{e}}_{\theta }\left(t\right)+k_{1}e_{\theta }\left(t\right)+k_{2}\int_{0}^{t}e_{\theta }\left(\tau \right)d\tau \end{array}$$

$k_{1}$ and $k_{2}$ are the parameter of the PID controller. The above controller can be converted to following discrete time controller for digital implementation,

$$\begin{array}{c} \sigma \left(k\right)=\left[\frac{e_{\theta }\left(k\right)-e_{\theta }\left(k-1\right)}{T_{s}}\right]+k_{1}e\left(k\right)+k_{2}\sum_{i=0}^{k}e_{\theta }\left(k\right)T_{s} \end{array}$$

and the deadband is added as follow,

(17) $$\begin{array}{c} u\left(k\right)=\left\{\begin{array}{cc} \sigma (t) & \mathrm{if}\;|\sigma (t)|>\epsilon \\ 0 & \mathrm{if}\;|\sigma (t)|<\epsilon , \end{array}\right. \end{array}$$

The experimental results are shown in Figure 8d. The figure shows the response of the system for two different values of parameters, $k_{1}$ and $k_{2}$. To present a fair comparison, we tuned the values of the PID parameters using the Ziegler–Nichols algorithm [56]. It can be seen that the response of the system in both cases shows overshooting. The first response (shown in red) corresponds to the parameter values: $k_{1}=1,\;k_{2}=1.5$, shows large overshoot as compared to the second response (shown in blue), which corresponds to $k_{1}=1.5,\;k_{2}=0.5$. In both cases, the bending angle overshoot to a maximum angle of about −4 degrees. The PID response is comparative to the response of Figure 8b, which shows approximately the same overshoot. However, the response of the PID controller is worse, then the SMC impedance controller in Figure 8c. The histograms of bending angles inside Figure 8d. The spread of histogram also affirms that the response is similar to Figure 8b. The presented experimental establishes the superiority of the proposed controller against the current state-of-the-art in SMC control for pneumatic soft robots.

## 6. Conclusions

In this paper, we presented an impedance control scheme for pneumatic soft robots. A nonlinear discrete-time sliding-mode controller based on impedance control was used to enforce the desired impedance dynamics on a soft pneumatic robot. The proposed controller automatically calculates the controller parameter based on the required impedance of the soft robot. It is in contrast with the traditional work on soft robotics, which uses manual parameter tuning to tune the parameters. The stability analysis of the proposed controller is also presented and proved that the controller is asymptotically stable. The 3D-design of our 6-chambered soft parallel robot was also presented along with the design and construction of the experimental platform used to conduct the experiments. It was proven through theoretical analysis and extensive comparative experimentation with the state-of-the-art PID controller that the proposed control scheme shows an outstanding performance in regulating the dynamic behavior of the soft pneumatic robot and suppressing the undesirable dynamic behavior, e.g., overshooting and vibrations. It is worth mentioning that this is the first work to apply the impedance control scheme to the recently developing field of soft robots, the inherent uncertainty of which makes the extension from rigid-body systems to soft-body ones a challenging topic.

## Data Availability

The raw data supporting the conclusions of this article will be made available by the authors on request.
